# Assessing Bioconcentration and Biotransformation of BDE-47 In Vitro: The Relevance of Bioavailable and Intracellular Concentrations

**DOI:** 10.3390/jox15030093

**Published:** 2025-06-16

**Authors:** Paloma De Oro-Carretero, Jon Sanz-Landaluze

**Affiliations:** Department of Analytical Chemistry, Faculty of Chemical Science, Complutense University of Madrid, Avenida Complutense s/n, 28040 Madrid, Spain; pdeoro@ucm.es

**Keywords:** bioaccumulation, biotransformation, in vitro, C_free_, IVIVE, internal concentration

## Abstract

The development of alternative methods that link cellular and predictive toxicity to high-level toxicity is a key focus of current research within the framework of the 3Rs in animal experimentation. In this context, this study aimed to evaluate the previously developed in vitro approach using the zebrafish liver cell line (ZFL) for assessing bioaccumulation and biotransformation of the compound BDE-47, which is more hydrophobic than phenanthrene, and is the compound used in the previous study. For this purpose, experimentally, the internal concentrations in the cells (C_cell_) and the exposure medium of both BDE-47 and its main metabolites were quantified at different exposure times by GC-MS. Additionally, the free bioavailable concentration (C_free_) was determined with a solid-phase microextraction (SPME) experiment. With the aim of refine models, C_cell_ and C_free_ were also estimated using a predictive chemical distribution model (MBM). Bioconcentration factors (BCFs) were determined by relating all these values, as well as by toxicokinetic fitting and by in vitro–in vivo extrapolation modelling (IVIVE). The results showed a high concordance with the values obtained in vivo. Moreover, the study highlighted the importance of experimentally determining C_free_ and C_cell_, as the predicted values can vary depending on the chemical, thereby influencing the BCF outcome. This variation occurs because models do not account for the absorption and biotransformation kinetics of the compounds. The data presented may contribute to refining predictive models.

## 1. Introduction

Understanding the behavior of chemicals in the environment throughout their life cycle, including bioaccumulation and biotransformation processes, is an essential requirement in ecotoxicological risk assessment and regulatory decision-making [1]. The increasing presence of xenobiotic compounds in the environment, along with the growing demand for 3R (Replacement, Reduction and Refinement) assays, has encouraged the research and design of ethical, fast and economically viable evaluation methodologies [2]. Therefore, emphasis is being placed on the development of alternative fish embryo testing (FET) [3], new approximation methods (NAMs) based on in vitro systems with cell lines [4] and in silico computational modelling and in vitro/in vivo extrapolation models [5].

Within the field of bioaccumulation assessment, Sanz-Landaluze et al. [6] obtained strong correlations of the bioconcentration factor (BCF) using zebrafish (*Danio rerio*) eleutheroembryos and those obtained using adult fish according to the Organization for Economic Co-operation and Development (OECD) guideline 305 [7] for a wide variety of compounds with different physico-chemical properties. The OECD 2018a guideline [8], referring to the methodology established by Nichols et al. [9], which determines BCF using empirical predictive regression and in vivo extrapolation of in vitro intrinsic clearance (IVIVE), determined according to the OECD 319 guideline [10,11], has shown great potential for BCF prediction. However, it has practical limitations for chemicals metabolized at low rates, due to the use of rainbow trout hepatocyte systems (RT-HEP) and rainbow trout liver subcellular fraction S9 (RT-S9), with assays limited to 4 h. Authors concluded that further studies and different substances are needed to expand the range of applicability of the method. De Oro-Carretero and Sanz-Landaluze evaluated the mentioned IVIVE model with the use of different in vitro systems based on hepatic and/or zebrafish cell lines, allowing assays of up to 72 h, resulting in the use of zebrafish liver cells (ZFL) being a promising approach for the determination of BCF in a quick, cheap and non-experimental way [12]. In addition, the necessity of the determination of the bioavailable concentration (C_free_) for the cells was demonstrated for a better approximation. This concentration was determined using the mass balance model (MBM) of the different compartments of an in vitro system [13], based on physico-chemical properties of the compounds. It has been shown that for substances with certain physico-chemical properties (more hydrophobic and non-neutral), the modeled C_free_ value may differ from the experimental bioavailable concentration and nominal concentration [14]. Consequently, it is imperative to conduct additional studies on the proposed approach using compounds with varying physicochemical characteristics and reduced metabolization rates.

Polybrominated diphenyl ethers (PBDEs) have been banned for several decades because of their known endocrine toxicity [15]; however, due to their persistent properties [16], the new emerging generation [17], and their metabolites, they continue to be the focus of ecotoxicological study. In this work, the compound 2,2′,4,4′- tetrabromodiphenyl ether (BDE-47) was selected because of its hydrophobic character (log K_ow_ 6.81 [18]), but within the working range of the IVIVE model (log K_ow_ between 3 and 8), and with slower metabolization kinetics, in order to (i) re-evaluate the IVIVE model with the ZFL cell line, (ii) evaluate the determination of C_free_ for BCF approximation with our published in vitro approach, and (iii) evaluate its in vitro biotransformation. The BDE-47 and their main metabolites (BDE-28, hydroxylated and methoxylated compound) concentrations inside the cell (C_cell_) and in the medium (C_medium_) were determined using the quick and simple method developed in a previous work [19]. Grasse et al. [20] studied the impact of biotransformation and absorption kinetics on the deviation of MBM model predictions with respect to the internal concentrations of organic compounds in zebrafish embryos for BCF prediction. Therefore, results obtained in these experiments were used to study the deviation of the concentration inside the cells (C_cell_) obtained by MBM and experimentally for the in vitro determination of BCF. In the same way, the C_free_ values obtained by MBM and those obtained experimentally by a solid-phase microextraction (SPME) experiment [21] were compared. These new results were also compared and discussed together, in this work, with those obtained with the same methodology in the previous study with the compound phenanthrene to provide information on different substances with different behaviors in in vitro systems and in the environment, to help refine the models. Therefore, all references and data found for phenanthrene in this manuscript were either taken from the previous study or calculated from the data reported in this one [12].

## 2. Materials and Methods

### 2.1. Reagents and Samples

Solid standard of BDE-47 was purchased from Sigma-Aldrich (Madrid, Spain). Commercial standards of triclosan (TCS) in acetone, and BDE-28 and BDE-99 in methanol were purchased from Dr. Ehrenstorfer GmbH (Augsburg, Germany); individual standards of 6-MeO-BDE-47, 5-MeO-BDE-47, 3-MeO-BDE-47 in methanol, 5-OH-BDE-47, 3-OH-BDE-47 and 2′-OH-BDE-28 in acetonitrile were supplied by AccuStandard Inc. (New Haven, CT, USA). The derivatization reagent N-tert-butyldimethylsilyl-N-methyltrifuoroacetamide (MTBSTFA) + 1% tert-butyl-methylchlorosilane was supplied by Sigma-Aldrich (Madrid, Spain). High-quality analytical solvents methyl tert-butyl ether (MTBE), dimethyl sulfoxide (DMSO), and hexane were purchased from Scharlab (Barcelona, Spain). Poly (dimethylsiloxane) (PDMS) and Polyacrylate (PA) fibers were supplied by Polymicro Tachnologies, Inc. (Phoenix, AZ, USA).

From Thermo Fisher Scientific (Madrid, Spain), Dulbecco’s Modified Eagle Medium (DMEM), Antibiotic/Antimycotic (A/A), TrypLE™ Express, Phosphate Buffered Saline (PBS), Fetal Bovine Serum (FBS), and Trypan Blue 0.4% were obtained. Sigma-Aldrich (Madrid, Spain) supplied bovine insulin, mouse epidermal growth factor (EGF), and 3-(4,5-dimethylthiazol-2-yl)-2,5-diphenyltetrazolium bromide (MTT). The heat-inactivated trout serum was supplied by the Institute of Biotechnology and Biomedicine (Barcelona, Spain). The American Type Culture Collection (ATCC, Manassas, VA, USA) provided the zebrafish liver cell line (ZFL).

ZFL cells were maintained at 28 °C in a humidified atmosphere containing 5% CO_2_, using DMEM medium enriched with 10% FBS, 5% antibiotics/antimycotics (A/A), 50 ng/mL EGF, 0.01 mg/mL bovine insulin, and 0.5% heat-inactivated trout serum, following the protocol described by Brandts et al. [22]. The medium was changed every 2 or 3 days and cells passed at 70% confluence.

### 2.2. Instrument and Apparatus

A Vibra cell VCx130 ultrasound probe from Sonics & Materials Inc., (Newtown, CT, USA) with a 2 mm-diameter titanium microtip and a 130 W high-frequency generator at 20 KHz, Eppendorf 5415R microcentrifuge (Hamburg, Germany), a Genie-2 vortex mixer from Scientific Industries and X50S metal carbide technical nitrogen (Barcelona, Spain) were used for extraction procedures. Incubator-genie from Scientific Industries. CO_2_ incubator (MIDI 40), glass and plastic P100 culture plates, 96-well plates and the Multiskan Sky High detector were purchased from Thermo Fisher Scientific (Madrid, Spain).

Instrumental analysis was carried out using an Agilent 7890A Series gas chromatograph, equipped with an HP 7683B autosampler, a microelectron capture detector (μECD), and an HP 5975C VL MSD mass spectrometry detector (Agilent Technologies, Madrid, Spain). Separation was achieved on a ZB-5 capillary column (30 m × 0.25 mm I.D., 0.25 μm film thickness) composed of 95% polydimethylsiloxane.

### 2.3. Cell Viability Assay

Cell viability was determined by MTT assay. A total of 10,000 cells/well were seeded in 96-well plates under culture conditions for 24 h. Then, the culture medium was replaced by contaminated medium (200 µL) with range of concentrations of BDE-47 (0.2–10 mg·L^−1^). Three independent replicates with the same experimental conditions, a control group (cells without pollutant) and a blank group (only cell culture medium) were performed. After 72 h, 20 µL of MTT solution (5 mg·mL^−1^) was added to provide a final concentration of 0.5 mg·mL^−1^ and incubated for 4 h. Formazan produced in the MTT assay was dissolved using 100 μL DMSO, and absorbance was recorded at 595 nm. The results have been subsequently used for the design of the bioaccumulation and biotransformation experiment.

### 2.4. Cell Exposure for Bioaccumulation and Biotransformation Evaluation

A total of 2 × 10^6^ ZFL cells were plated in P100 glass dishes and incubated for 24 h under standard culture conditions. Subsequently, the culture medium was replaced with a contaminated medium, which, according to the cell viability assay, maintained viability above 70% by the end of the exposure period (Section 2.3). Cells were exposed to 1.8 and 2.6 mg⋅L^−1^ for 24, 48 and 72 h, in two independent experiments. Parallel experiments were conducted under identical conditions to assess losses: one set included cells without contaminated medium, and another consisted of contaminated medium without cells (“no cells”). The obtained pellets were collected from all exposure time, dried under a gentle stream of nitrogen and weighed on a precision analytical balance (±0.00001 g). Finally, the dried cell pellets and their corresponding medium collected at all exposure times, including the starting medium (“t = 0 h”), were stored at −20 °C until analysis.

### 2.5. Experimental Determination of C_cell_ and C_medium_

Extraction of BDE-47 and its metabolites was performed simultaneously for each of the samples, cells, and exposure media. Different solvent mixtures were tested as shown in Figure S1. Finally, 500 μL of exposure medium was extracted with 500 μL of hexane: MTBE mixture (1:1) shaken vigorously with a vortex for 30 s. For cells, the pellet obtained (~5 mg) was treated with same solvents but assisted with an ultrasound probe for 1 min at 30% of amplitude and a pulse on/off, with 2 s/5 s for cell samples. The internal standards (IS) BDE-99 and TCS had been previously added. The mixture was centrifuged for 10 min at 10,000 rpm. The organic phase was separated, and the extraction step was repeated. Remaining extracts were evaporated to dryness under a gentle stream of nitrogen and reconstituted in 200 μL isooctane. A total of 80 μL of the volume obtained was derivatized with 20 μL of MTBSTFA at 70 °C for 30 min in the oven and injected into GC-μECD-MS for the OH-BDE quantification. PBDEs and MeO-BDEs were determined by direct injection of the remaining volume of isooctane extracts into the GC-µECD-MS, because PBDEs are not stable after the derivatization process. Due to the difference in concentration in the samples of the metabolites and BDE-47, a double determination was performed and diluted with isooctane for the detection of the latter, so that it was interpolated on the same calibration curve.

The GC–MS-µECD system was used for the separation and determination of BDE-47 and its metabolites. The MS was used for the identification and the µECD for their quantification. Both detectors operate simultaneously, thanks to the use of a flow splitter placed at the end of the chromatographic column. Sample (1 μL) were injected in splitless mode at 280 °C. Helium was used as carrier gas at a constant pressure of 22 psi. Column temperature was programmed to increase from 130 °C (1 min) to 222 °C (1 min) at a rate of 90 °C·min^−1^, then to 245 °C (1 min) at 1 °C·min^−1^ and finally to 280 °C (8 min) at 45 °C·min^−1^. The μECD temperature was set at 320 °C and nitrogen was used as makeup gas (30 mL·min^−1^). The flow ratio of the splitter was 1:1, thanks to the helium input and the restrictor length of each detector, according to the manual supplied by Agilent Technologies [23]. m/z values for the SIM program are collected in Table S1. All limits of detection, quantification and recoveries are shown in Table S2.

### 2.6. Data Analysis for C_cell_ and C_free_ Prediction with MBM

C_cell_ and C_free_ were determined for the BDE-47 using Fisher et al.’s mass balance model (MBM) [13]. In brief, the test system parameters for the experiment (Table S3) and BDE-47 distribution parameters (Table S4) estimated by the UFZ-LSER Calculation Partition System tool [24] were first entered into the MBM spreadsheet. Although this model includes preset parameters for various cell lines, the input fields for cell-line type and culture medium were left empty for ZFL, and generic parameters were applied instead. Then, data for fractions of cell (*f*_cell_), total medium (*f*_medium_), cell membrane (*f*_mem_) and the water phase of the medium (*f_w,_*_medium_) compartment were output (Table S5) and applied to each experimentally determined BDE-47 C_medium_ to estimate the C_free, MBM_ and C_cell, MBM_ at each exposure time.

### 2.7. Experimental Determination of C_free_

In parallel with the exposure experiment for the evaluation of bioaccumulation and biotransformation described in the previous sections, an experiment was performed to estimate experimentally the free bioavailable concentration (C_free,exp_) in the test medium. For this purpose, an experiment was designed to simulate the test conditions, and C_free_ was determined using solid-phase microextraction (SPME), following the fiber method developed by Henneberger et al. [21].

PDMS-coated glass microfibers (30 µm thick, 13.19 µL/m coating volume) were cut into 1.5 cm pieces and cleaned in methanol for more than 24 h prior to the experiment, with the solvent replaced at least three times. The cleaned and dried fiber was placed in a glass vial with 1 mL of sample exposure medium containing 1.8 and 2.6 mg·L^−1^ BDE-47. Three replicates were prepared for each condition. A portion of the initial sample was stored at −20 °C until analysis. The vials were then sealed and incubated with rotation at 28 °C for 72 h until equilibrium of BDE-47 distribution among the fiber, medium, and binding to the predominant serum protein of the culture (Bovine serum albumin, BSA) was reached [25]. Once at equilibrium, the fibers were extracted from the vials and desorbed with 400 µL isooctane for 24 h with rotation. Finally, the extract was analyzed by GC-MS-µECD, as described in Section 2.5. The concentration of BDE-47 in the initial sample (C_med,0h_) and in the resulting media after equilibration (C_med,eq_) was determined according to the procedure described in Section 2.5, to confirm the actual mass balance.

Since this study evaluates and discusses the behavior of BDE-47 in comparison to observations from a previous study with phenanthrene, as explained in the introduction section, the experimental C_free_ of phenanthrene was also determined, as it had not been measured previously. In this case, the difference in the process was that PA fibers (36 µm thick, 16.71 µL/m coating volume) were used. Samples of 10 and 20 mg·L^−1^ PHE were prepared, and the fibers and samples were incubated for 24–48 h until equilibrium was reached [26,27], and desorbed with hexane for 3 h.

To determine the free concentration, concentration in the SPME fiber (*C_fiber_*) was first estimated using Equation (1) from the concentration measured in the desorption extract (C_des_), the desorption volume (*V*_des_) and the coating volume of the fiber (V_fiber_). This volume was estimated according to the coating volume and the length of the fiber.

(1) $$C_{fiber}=\frac{C_{des}\cdot V_{des}}{V_{fiber}}$$

Next, *C*_free,exp_ was determined at the time before the SPME fiber was added to the system, according to Equation (2). For this, the concentration measured in the fiber equilibrated with the medium (PDMS-BDE-47-BSA or PA-PHE-BSA), the concentration measured in the initial sample (C_med,0h_), and the partition constant of the equilibrium formed between the fiber and the medium (log D_PDMS/medium_; BDE-47 = 5.84 [25]; log D_PA/medium_; PHE = 6.69 [26]) were used.

(2) $$C_{free}=\frac{C_{med,0h}\cdot V_{sample}}{D_{fiber/medium}\cdot \left(\frac{C_{med,0h}\cdot V_{sample}}{C_{fiber}}-V_{fiber}\right)}$$

### 2.8. Data Analysis for BCF Determination

#### 2.8.1. BCF Determination by In Vitro Experiment

The first quantitative estimation of BCF was carried out according to the definition of BCF in the official OECD 305 guideline [7]; that is, the ratio between the chemical concentration in the cells (C_cell_) and the chemical concentration of the surrounding medium (C_medium_). As explained earlier, C_cell_ can be determined experimentally (C_cell,exp_) or by MBM (C_cell,MBM_). Both these terms can be related to the experimentally determined concentration in the medium (C_medium_), the free concentration experimentally determined (C_free,exp_) or the free concentration estimated using MBM (C_free,MBM_). The different BCFs obtained from combinations of these values were determined, as shown in Equation (3).

(3) $${BCF}_{exp}=\frac{C_{cell,exp}}{C_{medium}};\;{BCF}_{Cfree}=\frac{C_{cell,exp}}{C_{free}};\;{BCF}_{MBM}=\frac{C_{cell,MBM}}{C_{free}}$$

The second model to estimate BCF was based on the first-order toxicokinetic model (Equations (4) and (5)), applying mathematical fitting of the experimental concentration values to the theoretical model describing the chemical uptake process [28,29].

(4) $$C_{cell}=\frac{k_{1}}{k_{2}}\cdot C_{W}\left(1-e^{-k_{2}t}\right)$$

(5) $${BCF}_{k}=\frac{C_{cell}}{C_{w}}=\frac{k_{1}}{k_{2}}$$

where C_w_ is the medium concentration, C_cell_ is the concentration inside cells at any time, t is the exposure time (h), and k_1_ (L·kg^−1^·h^−1^) and k_2_ (h^−1^) are the first-order uptake and elimination rate, respectively. Therefore, the BCF was calculated considering the experimental C_cell_ data (C_cell,exp_) with respect to the C_medium_ (BCF_k,Cexp/Cw_) and with respect to the C_free_ (BCF_k,Cexp/Cfree_), and considering the C_cell_ values obtained with the MBM with respect to C_medium_ (BCF_k,CMBM/Cw_) and with respect to C_free_ (BCF_k,CMBM/Cfree_).

#### 2.8.2. BCF Determination by IVIVE Model

One of the objectives was to assess whether cell lines can be used within the BCF-IVIVE model, relying on intrinsic clearance in vitro, to estimate the biotransformation rate which is subsequently extrapolated to adult fish. This approach is based on the model developed by Nichols et al. [9] and is endorsed by the OECD guideline 319A [10] and OECD 2018a [8]. Briefly, the log10-transformed substrate experimental concentrations in the medium (C_med_) were plotted against time, and the first-order elimination rate constant (k_e_, h^−1^) was determined as −2.3 × slope of the log-linear decline. Black et al. [30] indicate that a correction to the elimination rate constant (k_e_) should be applied when the following conditions are met: (i) abiotic losses from volatilization, calculated by comparing the recovery of negative control samples (“no cells”) at each exposure time to the initial time, fall outside the acceptable range of 80–120%, (ii) the loss process follows first order kinetics, and (iii) the ratio of k_e_ and the volatilization rate constant (k_volat_) is below 10 (k_e_/k_volat_ < 10). Then, the constant corrected for losses due to volatilization (k_e,loss_) was determined as −2.3 × the difference of the slopes of the sample and the loss control of the log-linear descent. The in vitro constant was then extrapolated to obtain an in vivo metabolization constant (k_MET_). This was done using experimental cell concentration (C_HEP_, 10^6^ cells/mL) and default parameters derived from rainbow trout. In this study, in addition to estimating BCF with the official model parameters derived from rainbow trout (BCF_IVIVE,RT_), BCF was also estimated with the literature-derived parameters for zebrafish (BCF_IVIVE,ZF_), due to the use of ZFL cells. These exchanged data are shown in Table S6. Finally, the BCF was estimated by the ratio of concentration in the organism to concentration in the medium, following the standardized model. The concentration in the organism was predicted using the one-compartment model given by Arnot and Gobas [31], which includes the rate constants describing chemical uptake (k_1_), loss through the gills (k_2_), fecal egestion (k_E_), growth rate (k_G_) and metabolization rate (k_MET_). Except for k_MET_, which is estimated experimentally in vitro, the rest are only dependent on the log K_OW_ of the compound. On the other hand, due to the use of cell lines, the free concentration estimated was introduced instead of the total concentration in the medium (C_W,TOT_).

## 3. Results and Discussion

### 3.1. Cell Viability

The dose–response fit is shown in Figure S2. Viability decreased by 30% and 50% with exposure to 0.45 and 2.03 mg·L^−1^ BDE-47, respectively. Yang and Chan also observed similar values of 1.60 and 1.26 mg·L^−1^ LC_50_ (24 h) and LC_50_ (96 h), respectively, using the Alamar Blue viability test for ZFL cells [32]. It is important to consider that these studies employed 10% FBS, which influences the bioavailable concentration to the cells and, consequently, impacts their viability. Therefore, for the calculation of a real value, comparable to studies that do not use such a high composition of supplements in the medium that could interact with BDE-47, the bioavailable concentration should be considered. When applying the percentage of C_free_ obtained in this study (Section 3.2) to the LC_30_ and LC_50_ concentrations obtained, values of 0.55 µg·L^−1^ and 0.12 µg·L^−1^, respectively, were estimated. Therefore, the MTT assay was performed to estimate the appropriate exposure concentration for bioaccumulation and metabolization experiments, ensuring that cell viability exceeds 70% after 72 h of exposure. Therefore, finally, it was decided to carry out the experiments at 1.8 mg·L^−1^ of BDE-47. Another experiment was performed at a concentration of 2.6 mg·L^−1^ of BDE-47 to find higher concentrations of metabolites.

### 3.2. Measured Concentrations of BDE-47 in the Culture Medium (Total and Free) and Inside Cells (Experimentally and by MBM)

**Figure 1:**
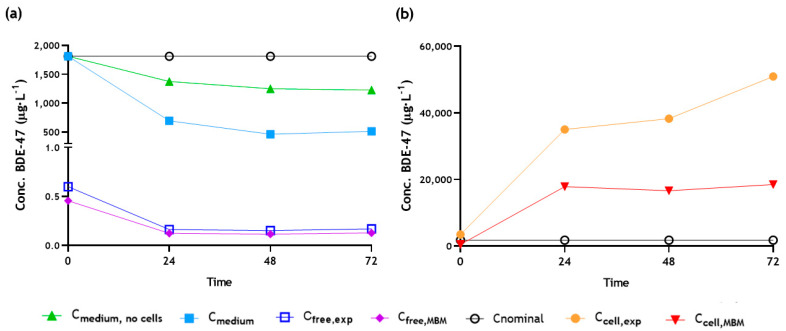
(**a**) Measured values of C_medium_, C_medium, no cells_, C_free,exp_ and C_free,MBM_ and (**b**) C_cell,exp_ and C_cell,MBM_ for BDE-47 at different exposure times in the 1.8 mg·L^−1^ experiment (C_nominal_).

Concentration of BDE-47 in the exposure media (C_medium_), in the control medium (C_medium, no cells_) and inside cells (C_cell,exp_) of each sample at 0 h, 24 h, 48 h and 72 h were determined experimentally, as described in Section 2.5. With the experimental value of C_medium_, the free concentration (C_free,MBM_) and cell concentration (C_cell,MBM_) were predicted by the mass balance model for each exposure time, as described in Section 2.6. The experimental C_free_ percentage was determined by the SPME fiber experiment, as described in Section 2.7, and applied to the C_medium_ to obtain the experimental C_free_ value (C_free,exp_) for each exposure time. All these results are collected in Figure 1 for the 1.8 mg·L^−1^. The same trends are found in the 2.6 mg·L^−1^ experiment (Figure S3).

C_medium_ decreased by an average of 79 and 33% over time, from the nominal or initial exposure concentration of the experiment of 1.8 and 2.6 mg·L^−1^, respectively. This decrease is due to the uptake of BDE-47 into the cells and abiotic losses from the in vitro system. The decrease in C_medium,no cells_ observed in the cell-free sample was 29 and 37% with respect to the nominal experimental concentration of 1.8 and 2.6, respectively. Because glass culture plates were used instead of plastic, preventing adhesion losses, and, given the volatile properties of BDE-47 (Henry’s law constant (K_H_) 1.79 ± 0.21 Pa·m^3^·mol^−1^ at 30 °C [33]), the observed losses are likely attributable to evaporation. In a previous study [19], a 14% volatilization loss with an initial exposure of 20 mg·L^−1^ for a HepG2 cell assay at 37 °C was observed (K_H_ 3.78a ± 0.18 at 30 °C [33]). Therefore, as noted in this work, this loss is dependent on the exposure concentration, so such losses must be quantified and considered for the determination of the metabolization constant with the substrate depletion approach in the IVIVE model (explained in Section 2.8.1), in order not to overestimate biotransformation and affect the BCF prediction, in accordance with Black et al. [30].

Free concentration estimated by MBM (C_free,MBM_) followed the same trend of decreasing with exposure time; however, the values were far below the total concentration present in the medium, with a free concentration of 0.03% of total concentration being observed. Dimitrijevic et al. [34] found stable values of the free concentration over time, but with a decrease from the initial concentration of 30% for the compounds studied with log P_ow_ > 2.59 and up to 50% for the compound tamoxifen (log P_ow_ = 6.84), determined by dialysis in samples of DMEM culture medium supplemented with 10% newborn calf serum and spiked to a final C_nom_ of 5 μmol·L^−1^ with 1% DMSO. Fisher et al. [13] reported that for hydrophobic compounds (log D_lipid/water_ = 7), the free concentration estimated by MBM was up to 4 orders of magnitude lower than the nominal concentration used in their experiments (C_nom_ = 4.86 mg·L^−1^ BDE-47). In a previous study, a free concentration of 1.7% of total concentration for phenanthrene (PHE) assays with ZFL cells was obtained. This difference in behavior between BDE-47 and PHE is due to BDE-47’s higher binding constant to the BSA protein of FBS (log DBSA/W = 5.53), compared to PHE (log D_BSA/W_ = 3.81). The BSA protein has three binding domains with two subdomains each [35], and both PAHs and PBDEs bind in the hydrophobic cavities (domains II and III). In the case of PBDEs, the BSA-water partition constant follows a linear trend with respect to the octanol–water partition constant; however, for PAHs this only occurs for those with log K_ow_ < 5 [25], due to binding limitations in the hydrophobic cavities of voluminous PAHs. PBDEs are bulkier but have higher affinity, and other specific binding mechanisms occur, i.e., electrostatic interactions due to the Br of their molecule [36]. This shows that the bioavailable concentration depends not only on the hydrophobicity of the compound, but also on the specific interactions with the serum of the medium and, therefore, it is important to study its behavior in the in vitro system to correctly determine the free concentration.

Regarding the C_free,exp_ determined by SPME experiment, as can be seen in Figure 1a and Figure S3a, the values obtained experimentally did not differ significantly from those obtained by MBM. Table 1 shows the C_free_ values determined, resulting in 0.06% and 0.08% of total concentration in the experiments with 1.8 and 2.6 mg·L^−1^ BDE-47, respectively. The Fisher et al. model [13] is well-defined for various neutral, anionic, cationic and multiprotic organic compounds across different media and cell types. It is not defined for the medium used for the ZFL cell line. The ZFL cell culture medium, in addition to the 10% FBS considered in the MBM, contains 0.5% rainbow trout serum. The small difference observed in C_free_ calculated from the experiment may be due to the presence of trout serum, which potentially causes a distributional shift in the equilibria present in the in vitro system. As shown in Table S7, trout serum has a higher amount of lipid-to-protein ratio compared to FBS, which may change the interaction dynamics. Given that the trout serum constitutes a small percentage relative to FBS, the interaction is primarily determined by the FBS concentration, resulting in a minimal difference in bioavailable concentration. Huchthausen et al. [14] reported that for substances exhibiting a non-linear binding to the medium components, the modeled C_free_ was underestimated, compared to the experimental one. This difference, although not significant, was observed also for phenanthrene (experimental C_free_ values shown in Table 1), so that bioavailable concentration percentages of 2.4 and 2.9% were obtained experimentally for the 10 and 20 mg·L^−1^ PHE exposures. Even though the MBM estimation yielded close results, experimental determination of C_free_ is strongly encouraged for substances or media types outside the model’s defined scope, as demonstrated in this work. Consequently, the BCF results shown in Section 3.3. have been determined using experimental C_free_.

Due to the bioaccumulative properties of BDE-47 [13,37], C_cell_ values exceeding the initial test concentration were obtained for both experimental and predicted data across all studied time points. Differences between experimental and MBM values were observed. The predicted values show a lower BDE-47 bioaccumulation and the reaching of steady state after 24 h of testing. In contrast, in our previous study on PHE, C_cell_ values estimated with the MBM were significantly higher than those obtained experimentally (the ratio of MBM vs. experimental was above 100%; Table S8). In the same way, Grasse et al. [20] showed that the experimental internal concentrations in zebrafish larvae of 79% of the substances studied (log D_lip/w_: −1.0–6.8) deviated less than 10-fold from the predictions and the rest by up to a factor of 90. These differences can affect the experimental or predicted bioconcentration factor, and consequently, the bioaccumulation assessment of chemicals, as discussed for BDE-47 with ZFL cells in the following section.

### 3.3. BCF Prediction Using ZFL Cells

#### 3.3.1. BCF Estimation Through In Vitro Assays Using ZFL Cells

BCF results obtained with different approaches explained in Section 2.8.1 are collected in Table 2. First, BCF calculation was based on the ratio between experimentally or MBM-determined cell concentrations and the medium (BCF_exp_ and BCF_MBM_) or the free dissolved concentration experimentally determined (BCF_Cfree,exp_) (Equation (3)), at each of the times studied. Next, BCF was calculated by fitting the experimental and predicted values using a non-linear regression (Figure S4) and a toxicokinetic model (BCF_k_, Equation (5)). The toxicokinetic parameters obtained are reported in Table S9.

Firstly, the bioconcentration factor (BCF) determined using the experimental total medium concentration (C_medium_) consistently yielded significantly lower values than when using the free bioavailable concentration, underestimating cellular bioaccumulation capacity (BCF_exp_ vs. BCF_Cfree,exp_). This observation aligns with our previous study on PHE, concluding that, in cell-based assays, where analytes may directly interact with constituents of medium, it is more accurate to calculate BCFs using the free concentration of the analyte. In contrast to results obtained for phenanthrene, BCF_k,Cfree_ and BCF_Cfree_ values resulted in non-matching values. This can be explained because, in the case of BDE-47, steady state was not reached in the ZFL cells during the time of the assay, as it has slower uptake kinetics. In such cases, according to OECD Guideline 305 [7], the first-order kinetic fit provides a more appropriate BCF estimate. Considering BCF_k Ccell exp/Cfree,exp_ and BCF_k Ccell MBM/Cfree,exp_ the resulting values were comparable to those obtained in a prior study, where the BCF of BDE-47 using zebrafish eleutheroembryos was determined: BCF_48 h_ values of 16,392 L·kg^−1^ and 4106 L·kg^−1^ and BCF_k_ values of 36,363 L·kg^−1^ and 7295 L·kg^−1^ when exposed to 1 µg·L^−1^ and 10 µg·L^−1^, respectively [38]. A literature review was carried out comparing values obtained in vivo with aquatic organisms and values obtained in silico based on quantitative models of structure–activity relationship (QSAR) models (Table 3). Since there are not many other references in the literature that discuss this topic for BDE47, these values will be used for comparison with the values obtained with the in vitro approach in this study. Briefly, Gustafsson et al. [39] and Vidal-Liñán et al. [40] estimated a BCF of 26,000 L·kg^−1^ and 10,900 L·kg^−1^, respectively, in *Mytilus galloprovincialis* (mussel). With the same degree of magnitude, Mhadhbi et al. [41] reported BCF values of BDE-47 in a young *Psetta maxima* of 24,125, 15,531, and 33,103 L·kg^−1^ at exposure of 1, 0.1, and 0.001 µg·L^−1^; Lebrun et al. [42] reported a lower BCF_k_ in amphipod crustacean *Gammarus pulex*, of close to 5000. The European Chemicals Bureau sets a value of BCF > 5000 in compounds with log K_ow_ between 5 and 8 [41]. Some agencies and authors have developed equations to predict the BCF value in aquatic organisms in relation to the octanol–water partition coefficient [43,44,45,46,47]. These methods are based on the capacity of the substance to be distributed in the lipid fraction, and provide a close approximation of the BCF prediction. Experimentally obtained BCF is preferable because the bioconcentration depends on the physiological responses of each organism, and the absorption and biotransformation kinetics of the compound [48,49].

In the same way, internal concentrations in organisms are often predicted by the mass balance model, assuming passive and non-specific uptake, without modulation of transformation processes, transport and uptake kinetics [50,51]. In this work, it is observed that the estimated BCF_k-CcellMBM/Cfree,exp_ values were lower than the BCF_k-Ccellexp/Cfree,exp_ determined with experimental C_cell_, because this internal value was higher than the one predicted by the Fischer et al. model [13] (Figure 1 and Figure S3). This discrepancy may arise because the model predicts an uptake that reaches steady state at 24 h, but BDE-47, as discussed previously, exhibits slow uptake kinetics in ZFL cells and, based on the experimental values, does not reach stability during the test (Figure S4). In contrast, in our previous study, the estimated C_cell_ values of phenanthrene using the MBM were significantly higher than those obtained experimentally (Table S8), resulting in a higher BCF_MBM_ value (1656 L·kg^−1^) compared to those obtained experimentally with ZFL cells (BCF_48 h_ = 393 L·kg^−1^ and BCF_k_ = 986 L·kg^−1^ on exposure to 10 mg·L^−1^ PHE). Unlike BDE-47, phenanthrene reached steady state in ZFL cells during the exposure time (72 h). Although both PAHs and PBDEs undergo biotransformation via cytochrome P450 phase I and II enzymes [52,53], the latter have slower biotransformation kinetics due to their bulkier structure and bromine substituents, which reduce their susceptibility to biotransformation and increase their potential for bioaccumulation. Since phenanthrene is rapidly metabolized and excreted, and these processes are not accounted for in the MBM model, it may result in an over-prediction of the internal concentration in the cell and, consequently, to a higher estimated BCF [20]. Thus, for some substances, the internal concentration estimated by current predictions deviates significantly from the experimental values when the bioaccumulative and biotransformation toxicokinetic of the compound are not considered and, consequently, this could lead to inaccurate assessments of chemical-specific ecotoxicity. Although current models offer rapid screening with a close approximation of substance bioaccumulation, further refinement is required. Incorporating detailed bioaccumulation and biotransformation toxicokinetic studies or supplementing with rapid, simplified assays, such as the in vitro approach presented herein, enhances alignment with experimental observations.

#### 3.3.2. Evaluation of ZFL Cells for BCF Prediction Using the IVIVE Model

BCF was also calculated using the IVIVE-BCF model developed by Nichols et al. [9] with rainbow trout and zebrafish default parameters (BCF_IVIVE,RT_ and BCF_IVIVE_,_ZF_ Table 2). For this methodology, the fits performed to determine the reaction rate by substrate depletion method and the rate constant of volatilization losses are shown in Figure S5. Following the criteria outlined by Black et al. [30], a correction was applied to the elimination rate constant (k_e_) in this study, because the percentage recovery of abiotic volatilization losses (% Rec. Vol.) fell outside the acceptable range of 80–120% (Table S10), the loss process followed first-order kinetics, and the ratio between k_e_ and the volatilization rate constant (k_volat_) was less than 10 (k_e_/k_volat_ < 10). Therefore, Table S11 shows the corrected reaction rate (k_e,loss_) used to determine IVIVE-BCF, as well as the main parameters calculated on the basis of this: in vitro intrinsic clearance (CL_IN VITRO, INT_), hepatic clearance (CL_H_) and whole-body metabolism rate (k_MET_).

The IVIVE-BCF approach of Nichols et al. [9] is a combination of in vitro and in silico assays, where the absorption (k_1_), elimination (k_2_), excretion (k_E_) and metabolization (k_MET_) constants are considered. However, while the metabolization constant is derived from in vitro experimental data, the absorption constants and internal organism concentration are estimated solely from the substance’s octanol–water partition coefficient, lacking experimental validation. Certainly, the BCF values obtained for BDE-47 using the IVIVE-BCF model (BCF_IVIVE_, Table 2) strongly match with in vivo values reported in the literature (Table 3), both of those calculated with zebrafish or rainbow trout default parameters. As discussed in Section 3.3.1, predicted intracellular concentrations can deviate significantly from experimental values for certain substances, due to variations in absorption kinetics, which are poorly defined when relying solely on K_ow_. De Oro-Carretero and Sanz-Landaluze [12] illustrated this phenomenon, wherein the IVIVE-BCF model, utilizing K_ow_-derived internal concentrations, yielded satisfactory PHE BCF values for ZFL and HepG2 cells. In contrast, the application of experimental cellular internal concentrations within the toxicokinetic model resulted in markedly different BCF values, reflecting the divergent uptake kinetics of the two cell lines. It is shown that, for some substances, it is important to consider the experimental study of the absorption kinetics to obtain a value closer to reality. Therefore, in addition to the BCF_IVIVE_ prediction based on model equations as established by Nichols et al. [8], in this work BCF_IVIVE,exp_ (Table 2) has been calculated in the same way, but using the values of the constants k_1_ and k_2_ obtained (Table S9) with the experimental values C_cell_ and C_free_, and adjusting to the units of the model and normalizing with the weight of the fish for extrapolation. Similarly, the values obtained were quite like those obtained in vivo.

With these results, extending our previous in vitro study with phenanthrene, the potential of the IVIVE-BCF model using the ZFL cell line is again demonstrated. This approach enables the evaluation of compounds with slower bioaccumulation and metabolization kinetics, without the need to use hepatocytes and, therefore, animal experimentation. An experimental design and an easy and simple analytical method previously developed [19] have been applied in this study, allowing us to determine the internal concentrations of the cells over time, to estimate the uptake constant experimentally. In this sense, it has been demonstrated that it is possible to incorporate it into the IVIVE-BCF model, as is the case with the determination of the metabolization constant, which, with the combination of extrapolation, can obtain satisfactory results for BCF.

### 3.4. Biotransformation of BDE-47 in ZFL Cells

This study, along with numerous others on this topic, has demonstrated the importance of studying biotransformation simultaneously with bioaccumulation for accurate BCF estimation [48,49]. Additional data on the biotransformation of model chemicals in in vitro systems is needed to understand their behavior and for refining predictive models [20].

During exposure of ZFL cells to BDE-47 at 1.6 mg·L^−1^ and 2.8 mg·L^−1^, concentrations of the metabolites 5-MeO-BDE-47 were detected in the cell samples, and the metabolites 5-OH-BDE-47 and 3-OH-BDE-47 in the medium. The ratios found (the ratio of metabolite concentration to free bioavailable concentration of BDE-47) at each exposure time are shown in Table 4. No concentrations of the mentioned metabolites were detected in the control samples with “no cells” and at the beginning of the experiment (“t = 0 h”), and no concentrations of the metabolites 6-MeO-BDE-47 and 3-MeO-BDE-47 above the limit of quantification were detected in any of the samples. A significant and increasing concentration over time of BDE-28 and 2′-OH-BDE-28 was detected in the cell and medium samples. BDE-28 is a major metabolite resulting from the loss of bromine at the ortho position [54] and, consequently, 2′-OH-BDE-28 is also a metabolite of BDE-47. However, impurities of BDE-28 can be found in commercial BDE-47 standards [55] and, indeed, quantifiable concentrations were found in the ‘t = 0 h’ and ‘no cells’ samples and, although always below 1% with respect to BDE-47, it cannot be differentiated whether the concentrations found in the samples were due to the biotransformation of BDE-47 or the bioaccumulation of BDE-28 itself, present as impurity. The results for BDE-28 and 2′-OH-BDE-28 were not considered in this study.

Hydroxylated metabolites (OH-BDEs) were detected in the medium samples, likely due to phase I detoxification reactions involving the introduction of a polar hydroxyl group, facilitating their excretion. Conversely, methoxylated metabolites, derived from the interconversion of OH-BDEs, were found predominantly in the cell samples, presumably because their lower polarity hinders cellular excretion [52]. Similar results were reported when exposing 20 mg·L^−1^ BDE-47 to HepG2 cells [19]. These metabolites can be coupled normally to endogenous compounds in phase II to form other intermediate metabolites. A mass balance (Equations (6) and (7)) has been performed, considering the experimentally determined concentrations of BDE-47 and its metabolites in the cells and the exposure medium at each exposure time (*xh*), as well as the losses due to the volatility of the compound (Equation (8)), and the recovery at the end of the test was estimated (Equation (9)). While acceptable recovery percentages exceeding 70% were achieved (Table 5), the development of a method for the in vitro identification and quantification of intermediate metabolites is crucial for future studies, enabling more comprehensive analysis and subsequent modeling.

(6) $$m_{Total}={mBDE47}_{0h}=m_{xh}\;$$

(7) $${\;\;\;\;m}_{xh}={{(mBDE47}_{medium})}_{xh}+{{(mBDE47}_{cells})}_{xh}{{+(mBDE47}_{loss})}_{xh}+({{\sum mMetab.}_{medium})}_{xh}+{{\sum mMetab.}_{cells})}_{xh}$$

(8) $${{mBDE47}_{loss}}_{xh}={\left({mBDE47}_{medium\;sample}\right)}_{xh}-{\left({mBDE47}_{medium\;no\;cells}\right)}_{xh}$$

(9) $$\;Recovery\left(\%\right)=\frac{m_{xh}}{{mBDE47}_{0h}}\cdot 100\;$$

Using previously obtained data in the exposure of BDE-47 to HepG2 cells and considering the estimated C_free_ percentage, the ratios of the metabolites found were calculated (Table 4), finding ratios very similar for both types of cells from human and fish [19]. All these results, together with those obtained previously with phenanthrene [12], confirms the metabolizing capacity of ZFL cells of compounds with slower kinetics.

In the comparison of in vitro and in vivo values, because in previously reported values with zebrafish eleutheroembryos only the metabolites inside the larvae were determined, not those in the exposure medium, therefore only 5-MeO-BDE-47 is comparable to the results with ZFL cells [38]. The estimated in vivo ratio (Table 4) was of the same order of magnitude as those obtained with the in vitro data. Lungu et al. [51] reported a strong correlation between ZFL cell-line assays, zebrafish embryo assays and in vivo adult zebrafish assays for toxicity effect measurements, but only when linked to modeled bioavailable concentrations. Thus, awaiting further testing with other types of substances, the results obtained show the viability of ZFL cells for biotransformation studies, opening the possibility of being used in IVIVE models for the determination of BCF.

## 4. Conclusions

This study aimed to re-evaluate our previously developed in vitro approach using a more hydrophobic compound with slower biotransformation kinetics: BDE-47 compared to phenanthrene. Our findings confirm the potential of using zebrafish liver (ZFL) cell lines and the determination of free available concentration (C_free_) for estimating bioconcentration factors (BCFs) within IVIVE frameworks. The experimental BCF values obtained using this in vitro system showed good agreement with those reported in vivo and from QSAR predictions, supporting its use as a reliable and animal-free alternative.

We also observed that internal concentrations estimated by mass balance models (MBM) may deviate from experimental values, particularly for substances with complex uptake or metabolic kinetics. This highlights the importance of integrating experimental data on absorption, internal concentrations, and metabolization, to improve model accuracy.

Finally, the analytical method and experimental design applied here proved to be rapid, cost-effective, and suitable for integration into IVIVE-BCF modeling. This contributes valuable insights toward refining predictive models and reducing the need for animal testing in environmental risk assessment.

## Figures and Tables

**Table 1:** Experimentally estimated bioavailable concentration (C_free,exp_) for BDE-47 and PHE under bioaccumulation and biotransformation experiment conditions.

	C_nom_ (mg·L^−1^)	C_free, exp_	% C_free, exp_
BDE-47	1.8	(0.4 ± 0.2) µg·L^−1^	0.06 ± 0.03
	2.6	(1.14 ± 0.04) µg·L^−1^	0.079 ± 0.003
PHE	10	(0.22 ± 0.02) mg·L^−1^	2.4 ± 0.2
	20	(0.52 ± 0.04) mg·L^−1^	2.9 ± 0.2

**Table 2:** BCF values (L·kg^−1^) obtained for BDE-47 using in vitro approaches with ZFL cell line.

C_nominal_ BDE-47	1.8 mg·L^−1^	2.6 mg·L^−1^
BCF_exp, 24 h_	50	51
BCF_exp, 48 h_	83	55
BCF_exp, 72 h_	100	80
BCF_Cfree,exp, 24 h_	2.2 10^5^	1.2 10^5^
BCF_Cfree,exp, 48 h_	2.5 10^5^	2.1 10^5^
BCF_Cfree,exp, 72 h_	3.0 10^5^	2.81 10^5^
BCF_MBM; 24 h, 48 h, 72 h_	1.4 10^5^	1.4 10^5^
BCF_k Ccell exp/Cw_	30	42
BCF_k Ccell MBM/Cw_	10	23
BCF_k Ccell exp/Cfree,exp_	74,300	63,435
BCF_k Ccell MBM/Cfree,exp_	8588	38,558
BCF_IVIVE, ZF_	13,885	14,926
BCF_IVIVE, RT_	7221	14,184
BCF_IVIVE, ZF, exp_	22,614	38,948
BCF_IVIVE, RT, exp_	26,306	48,635

**Table 3:** BDE-47 Bioconcentration factor values estimated from the literature and different QSAR models.

Reference	In Vivo Exposure/QSAR Equation	Life Stage	BCF (L·kg^−1^)
[38]	BCF_48 h_ (1 µg·L^−1^)	Zebrafish larvae	16,392
	BCF_48 h_ (10 µg·L^−1^)		4106
	BCF_k_ (1 µg·L^−1^)		36,363
	BCF_k_ (10 µg·L^−1^)		7295
[39]	BCF_k_ (0.31 ng·L^−1^)	Mytilus galloprovincialis (mussel)	26,000
[40]	BCF_k_ (8 µg·L^−1^)		10,900
[41]	BCF_k_ (1 µg·L^−1^)	young Psetta maxima	24,125
	BCF_k_ (0.1 µg·L^−1^)		15,531
	BCF_k_ (0.001 µg·L^−1^)		33,103
[42]	BCF_k_ (1 µg·L^−1^)	Gammarus pulex	5000
[43]	$\log BCF=-0.20\cdot \log{K_{ow}}^{2}+2.74\cdot \log K_{ow}-4.72\;$	Aquatic organism	46,200
[44]	$\log BCF=0.60\cdot \log K_{ow}-0.23$	Fish	7180
[45]	$\log BCF=0.86\cdot \log K_{ow}-0.4634$	Zebrafish larvae	247,000
[46]		Fish	13,600
[47]	Total water	Generic lab fish	11,100
	Dissolved water	Generic lab fish	30,800

**Table 4:** Biotransformation ratio between metabolites and BDE-47 concentration obtained in this work with ZFL cells and with HepG2 cells and zebrafish larvae obtained in our previous studies.

Model	5-MeO (Cells/Larvae Samples)		3-OH (Medium Samples)		5-OH (Medium Samples)	
C_nominal_ BDE-47	1.6 mg·L^−1^	2.8 mg·L^−1^	1.6 mg·L^−1^	2.8 mg·L^−1^	1.6 mg·L^−1^	2.8 mg·L^−1^
ZFL cells (24 h)	0.11	0.17	0.07	0.08	0.07	0.13
ZFL cells (48 h)	0.12	0.18	0.08	0.08	0.10	0.15
ZFL cells (72 h)	0.15	0.20	0.09	0.15	0.11	0.18
HepG2 cells (48 h)	0.10		0.13		0.2	
Zebrafish embryos (48 h)	0.06–0.13		-		-	

**Table 5:** Recovery (%) obtained in the mass balance at 1.8 mg·L^−1^ and 2.6 mg·L^−1^ of BDE-47 in ZFL.

Time Exposure	Experiment 1.8 mg·L^−1^	Experiment 2.6 mg·L^−1^
24 h	77	79
48 h	70	82
72 h	74	97

## Data Availability

No additional data.

## Supplementary Materials

The following supporting information can be downloaded at: https://www.mdpi.com/article/10.3390/jox15030093/s1, Table S1: Mass spectrometer conditions in SIM mode for BDE-47 and their metabolites; Table S2: Limits of detection (LODs), limits of quantification (LOQs) and recoveries for BDE-47 and its metabolites in cells and media samples; Table S3: Test system parameters for the experiment input in MBM spreadsheet and system information output from MBM; Table S4: Chemical parameters input in MBM spreadsheet estimated by UFZ-LSER Calculation Partition System (pH 7.4, 37 °C); Table S5: Chemical partitioning estimated by MBM spreadsheet; Table S6: Default parameters derived from rainbow trout and zebrafish for IVIVE-BCF estimation model; Table S7: Amount of protein and lipid content in fetal bovine serum (FBS) and rainbow trout serum used to supplement the ZFL cel-l line culture medium; Table S8: Ratio (in %) of the internal concentration estimated by MBM and experimentally for the BDE-47 experiment of this work and the PHE experiment in our previous work, in ZFL cells; Table S9. Toxicokinetic parameters calculated with the cell concentration values obtained experimentally or by MBM and C_free_ obtained experimentally for BDE-47 bioconcentration at 1.8 mg·L^−1^ and 2.6 mg·L^−1^; Table S10: Recovery of losses from volatilization (% Rec. Vol) when exposed to 1.8 mg·L^−1^ and 2.6 mg·L^−1^ at different exposure times. The percentages were calculated from the ratio of the concentration determined in the medium of the samples without cells (‘no cells’) from the different exposure times, to the initial dilution (time 0 h); Table S11: Input and calculated parameter by IVIVE-BCF model at 1.8 mg·L^−1^ and 2.6 mg·L^−1^ of BDE-47 using the zebrafish (ZF) and rainbow trout (RT) default parameters (shown in Table S5). The BCF BCF_IVIVE,RT_ and BCF_IVIVE,ZF_ are shown in Table 2; Figure S1: Recoveries obtained with the different extractants in medium samples of known concentration (5 µg·L^−1^); Figure S2: ZFL cell viability of different BDE-47 concentration at 72 h; Figure S3. (a) Measured values of C_medium_, C_medium, no cells_, C_free,exp_ and C_free,MBM_ and (b) C_cell,exp_ and C_cell,MBM_ for BDE-47 at different exposure times in the 2.6 mg·L^−1^ experiment (C_nominal_); Figure S4: First-order kinetic fit of the BDE-47 internal concentration in ZFL cells determined experimentally in the 1.8 (a) and 2.6 mg·L^−1^ (b) experiment; and by MBM in the 1.8 (c) and 2.6 mg·L^−1^ (d) experiment; Figure S5: Fits performed to determine the reaction rate (k_e_) by BDE-47 depletion in the 1.8 (a) and 2.6 mg·L^−1^ (b) experiment method and the rate constant of BDE-47 volatilization losses (k_e,volat_) in the 1.8 (c) and 2 mg·L^−1^ (d) experiment; References [8,9,12,13,24,56,57,58] are cited in the supplementary materials.
