# Supplementary material for: Assessing Bioconcentration and Biotransformation of BDE-47 In Vitro: The Relevance of Bioavailable and Intracellular Concentrations
Source: J Xenobiot. 2025 Jun 16;15(3):93. doi: 10.3390/jox15030093 (PMC12194305; doi:10.3390/jox15030093)
Supplement: Supplementary file 1 [file jox-15-00093-s001.zip › jox-3590224-supplementary.pdf]

Assessing Bioconcentration and Biotransformation of BDE-47 In Vitro: The Relevance of Bioavailable and Intracellular Concentrations

Paloma De Oro-Carretero and Jon Sanz-Landaluze *

Department of Analytical Chemistry, Faculty of Chemical Science, Complutense University of Madrid, Avenida Complutense s/n, 28040 Madrid, Spain
* Correspondence: jsanzlan@ucm.es; Tel.: +034-91-394-4322; Fax: +034-91-394-4329

Supporting information

Table S1. Mass spectrometer conditions in SIM mode for BDE-47 and their metabolites

Analyte	Retention time (min)	m/z	Initial scanning time (min)
BDE-28	11.00	248, 246, 406, 408	8 (solvent delay)
TMDS-TCS	12.92	290, 288, 218	12
BDE-47	17.51	326, 486, 484, 488	15
2’-OH-TBDMS-BDE-28	22.29	423*, 421*, 425, 81	20*
6-MeO-BDE-47	22.38	516, 514*, 518, 420	
3-MeO-BDE-47	24.28	356*, 516, 341, 514	
5-MeO-BDE-47	24.87	516*, 356, 358, 326	
BDE-99	27.33	404, 406, 564, 566	26
5-OH-TBDMS -BDE-47	30.92	502*, 504*, 500, 81	29*
3-OH-TBDMS -BDE-47	35.16	502, 474*, 266, 419	

* A joint window is established, as they elude close retention times, so that the majority m/z of each is monitored (marked in the table). TBDMS-X = tert-butyldimethylsilylated derivative

Table S2. Limits of detection (LODs), limits of quantification (LOQs) and recoveries for BDE-47 and its metabolites in cells and media samples

	LODs ($\mu g \cdot L^{-1}$)		LOQs ($\mu g \cdot L^{-1}$)		Recoveries (%)	
**Analyte**	**Cells**	**Medium**	**Cells**	**Medium**	**Cells**	**Medium**
BDE-47	1.65	2.69	8.63	8.96	80 ± 5	82 ± 9
BDE-28	0.005	0.001	0.016	0.002	100 ± 7	125 ± 9
2'-OH-BDE-28	0.002	0.001	0.006	0.004	75 ± 7	61 ± 9
6-MeO-BDE-47	0.01	0.30	0.02	0.99	67 ± 1	84 ± 9
3-MeO-BDE-47	0.001	0.001	0.005	0.002	81 ± 5	82 ± 9
5-MeO-BDE-47	0.005	0.002	0.017	0.007	90 ± 9	84 ± 7
5-OH-BDE-47	0.002	0.022	0.006	0.074	79 ± 5	114 ± 8
3-OH-BDE-47	0.002	0.001	0.008	0.003	83± 9	95 ± 2

Table S3. Test system parameters for the experiment input in MBM spreadsheet (Fischer et al. 2017) and system information output from MBM

	**ZFL**
**Test system parameters**	
Medium volume (µL)	10000
Type of base medium	DMEM Glutamax
Fraction of base medium (%)	90
Type of serum	FBS
Fraction of serum (%)	10
Cell line	-
Cell number at test start	5571000
**System information**	
Water content (%)	88.4
Protein content (%)	9.3
Lipid content (%)	2.3

Table S4. Chemical parameters input in MBM spreadsheet (Fischer et al. 2017) estimated by UFZ-LSER Calculation Partition System (pH 7.4, 37 °C) (Ulrich 2017).

**Compound**	**log $D_{BSA/water}$ [L/L]**	**log $D_{lipid/water}$ [L/L]**
BDE-47	5.53	6.70

Table S5. Chemical partitioning estimated by MBM spreadsheet (Fischer et al. 2017).

**Cell line**	$f_{w,medium}$ **(%)**	f_{cell} **(%)**	f_{medium} **(%)**	f_{mem} **(%)**
ZFL	0.02	7.88	92.12	6.16

Table S6. Default parameters derived from rainbow trout and zebrafish for IVIVE-BCF estimation model (OECD 2018)

	**Rainbow Trout**	**Zebrafish**
Modeled body weight (B_{wgM}) (g)	10[a]	0.5[c]
Modeled temperature (T) (°C)	12[b]	28[b]
Fractional liver weight (L_{FBW}) (g liver/g fish)	0.015[a]	0.033[d]
Liver hepatocyte content (L_{HEP}) (10^6 cells/g liver)	510[a]	56.1[e]

[a] (Nichols et al. 2013; OECD 2018)
[b] Experiment temperature
[c] (Castranova et al. 2011)
[d] (Cheng et al. 2016)
[e] Estimated

Table S7. Amount of protein and lipid content in fetal bovine serum (FBS) and rainbow trout serum used to supplement the ZFL cel- line culture medium.

**Serum**	**Protein content (g/L)**	**Lipid content (g/L)**	**Reference**
FBS	71.75	1.57	(Fischer et al. 2017)
Trout Serum	39.69	12.13	(Henneberger et al. 2020)

Table S8. Ratio (in %) of the internal concentration estimated by MBM and experimentally for the BDE-47 experiment of this work and the PHE experiment in our previous work (De Oro-Carretero and Sanz-Landaluze 2024), in ZFL cells.

**Ratio MBM/experimental (%)**	**BDE-47 experiment**		**PHE experiment**	
Time exposure / Nominal concentration	1.8 mg·L^{-1}	2.6 mg·L^{-1}	10 mg·L^{-1}	50 mg·L^{-1}
24h	51	73	491	510
48h	44	78	532	449
72h	36	66	421	402

Table S9. Toxicokinetic parameters calculated with the cell concentration values obtained experimentally or by MBM and C_{free} obtained experimentally for BDE-47 bioconcentration at 1.8 mg·L^{-1} and 2.6 mg·L^{-1}

	**Experiment 1.8 mg·L^{-1}**		**Experiment 2.6 mg·L^{-1}**	
	**k_1 (L·µg·h^{-1})**	**k_2 (µg·L^{-1})**	**k_1 (L·µg·h^{-1})**	**k_2 (µg·L^{-1})**
**$BCF_{K,Cexp/Cw}$**	1.20	0.04	2.96	0.17
**$BCF_{K,Cexp/Cfree}$**	3715	0.04	10784	0.17
**$BCF_{K,CMBM/Cw}$**	7.838	0.803	2.784	0.121
**$BCF_{K,CMBM/Cfree}$**	6896	0.803	4627	0.121

Table S10. Recovery of losses from volatilization (% Rec. Vol) when exposed to 1.8 mg·L^{-1} and 2.6 mg·L^{-1} at different exposure times. The percentages were calculated from the ratio of the concentration determined in the medium of the samples without cells ('no cells') from the different exposure times, to the initial dilution (time 0h).

**Time exposure / % Rec. Vol.**	**Experiment 1.8 mg·L^{-1}**	**Experiment 2.6 mg·L^{-1}**
24h	76	73
48h	69	63
72h	68	52

Table S11. Input and calculated parameter by IVIVE-BCF model (OECD 2018) at 1.8 mg·L^{-1} and 2.6 mg·L^{-1} of BDE-47 using the zebrafish (ZF) and rainbow trout (RT) default parameters (shown in Table S5). The BCF $BCF_{IVIVE,RT}$ and $BCF_{IVIVE,ZF}$ are shown in Table 2.

**Experiment**	**1.8 mg·L^{-1}**		**2.6 mg·L^{-1}**	
**Default parameters**	**ZF**	**RT**	**ZF**	**RT**
**Input parameters**				
$C_{w,TOT}$ ($C_{free,exp}$) (mg/L)	0.0006		0.0016	
C_{HEP} (10^6 cells/mL)	0.557		0.557	
$k_{e,loss}$ (h^{-1})	0.012		0.002	
**Calculated parameters**				
Binding correction term (f_U)	0.0495		0.0495	
$CL_{\mathit{IN\ VITRO},\ INT}$ (mL/h/10^6 cells)	0.0220	0.0220	0.0037	0.0037
CL_H (L/d/kg)	0.0482	0.1972	0.0080	0.0332
k_{MET} (d^{-1})	0.0022	0.0092	0.0004	0.0015
k_1 (L/kg/d)	2091	631	2091	631
k_2 (d^{-1})	0.006	0.002	0.006	0.002

Figure S1. Recoveries obtained with the different extractants in medium samples of known concentration (5 µg·L^{-1})

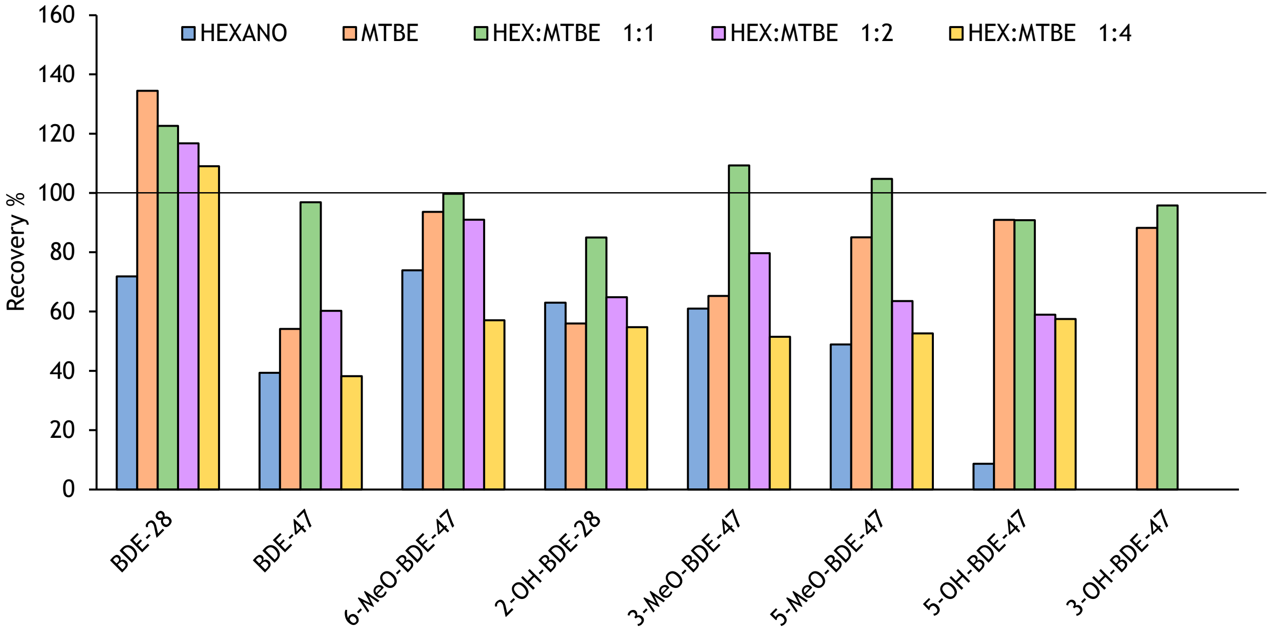

Figure S2. ZFL cell viability of different BDE-47 concentration at 72h

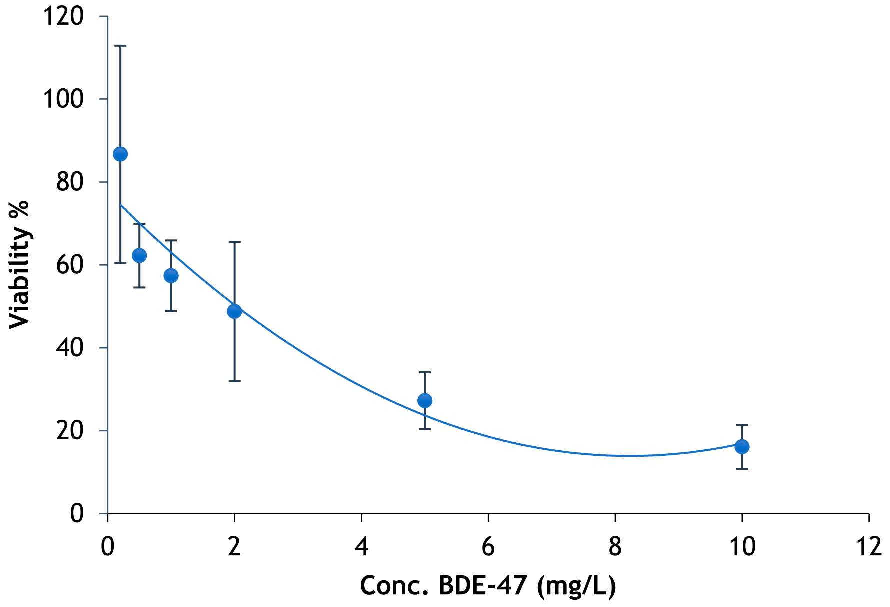

Figure S3. a) Measured values of C_{medium}, $C_{medium,\ no\ cells}$, $C_{free,exp}$ and $C_{free,MBM}$ and b) $C_{cell,exp}$ and $C_{cell,MBM}$ for BDE-47 at different exposure times in the 2.6 mg·L^{-1} experiment ($C_{nominal}$)

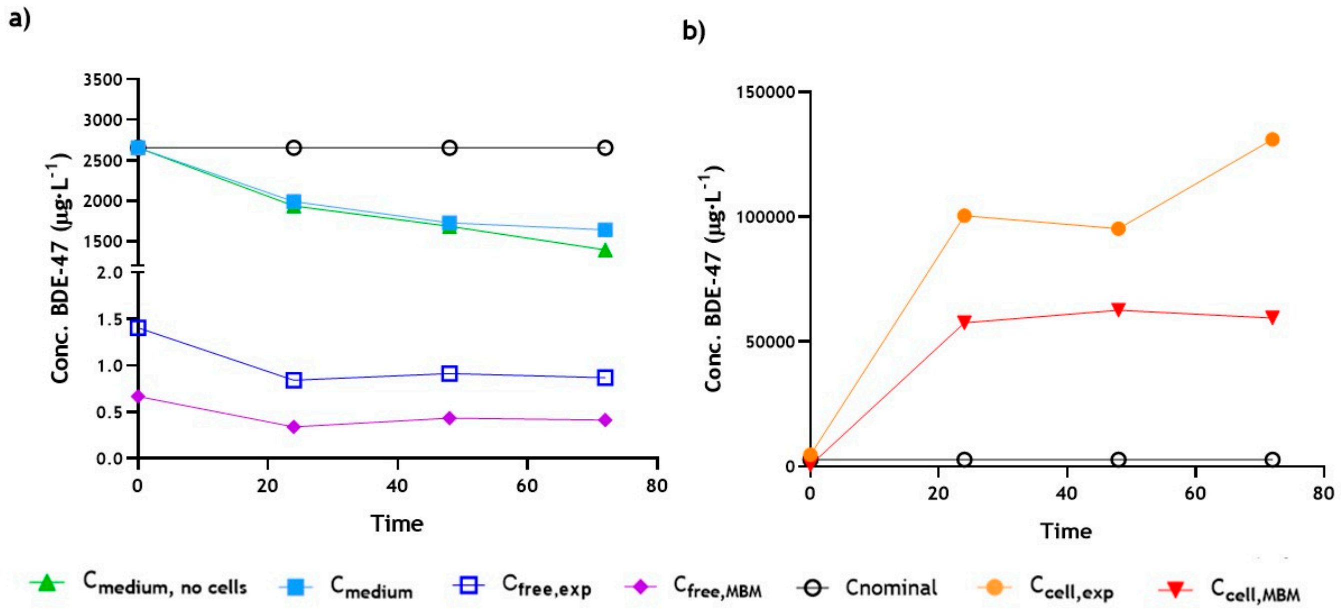

Figure S4. First-order kinetic fit of the BDE-47 internal concentration in ZFL cells determined experimentally in the 1.8 (a) and 2.6 mg·L^{-1} (b) experiment; and by MBM in the 1.8 (c) and 2.6 mg·L^{-1} (d) experiment.

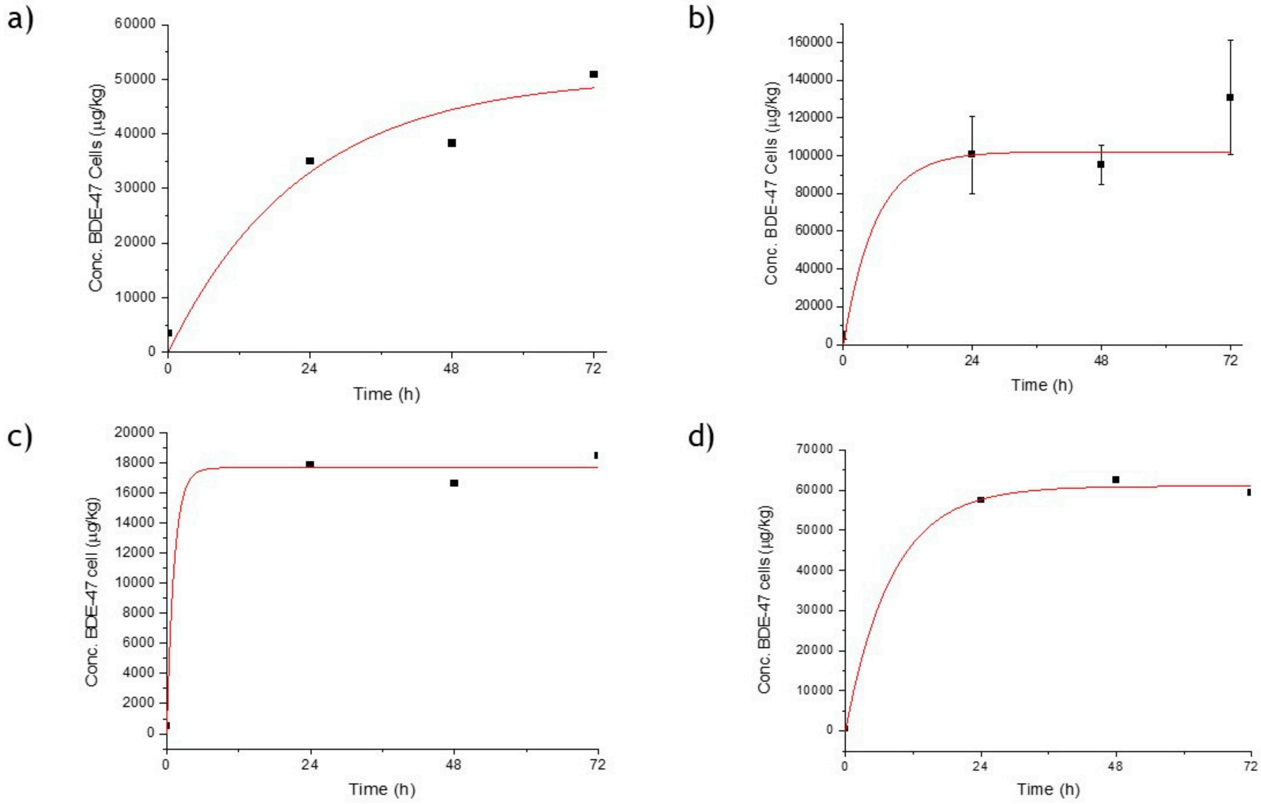

Figure S5. Fits performed to determine the reaction rate (k_e) by BDE-47 depletion in the 1.8 (a) and 2.6 mg·L^{-1} (b) experiment method and the rate constant of BDE-47 volatilization losses ($k_{e,volat}$) in the 1.8 (c) and 2 mg·L^{-1} (d) experiment

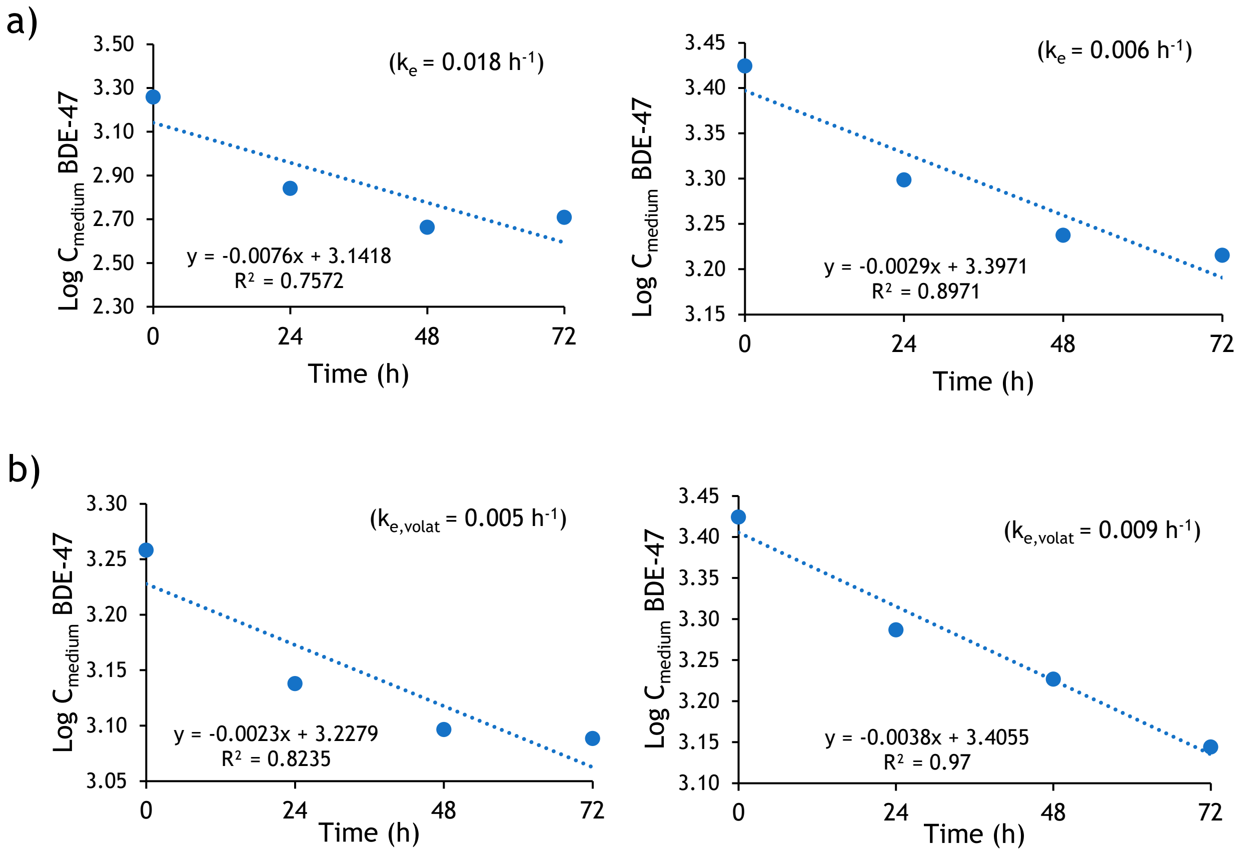

References

Castranova, Daniel, Angela Lawton, Christian Lawrence, Diana P. Baumann, Jason Best, Jordi Coscolla, Amy Doherty, Juan Ramos, Jenna Hakkesteeg, Chongmin Wang, Carole Wilson, James Malley, and Brant M. Weinstein. 2011. "The Effect of Stocking Densities on Reproductive Performance in Laboratory Zebrafish (Danio Rerio)." *Zebrafish* 8(3):141–46. doi: 10.1089/zeb.2011.0688.

Cheng, Delfine, Gerald J. Shami, Marco Morsch, Roger S. Chung, and Filip Braet. 2016. "Ultrastructural Mapping of the Zebrafish Gastrointestinal System as a Basis for Experimental Drug Studies." *BioMed Research International* 2016. doi: 10.1155/2016/8758460.

Fischer, Fabian C., Luise Henneberger, Maria König, Kai Bittermann, Lukas Linden, Kai Uwe Goss, and Beate I. Escher. 2017. "Modeling Exposure in the Tox21 in Vitro Bioassays." *Chemical Research in Toxicology* 30(5):1197–1208. doi: 10.1021/acs.chemrestox.7b00023.

Henneberger, Luise, Nils Klüver, Marie Mühlenbrink, and Beate Escher. 2020. "Trout and Human Plasma Protein Binding of Selected Pharmaceuticals Informs the Fish Plasma Model." *Environmental Toxicology and Chemistry* 41(3):559–68. doi: 10.1002/etc.4934.

Nichols, John W., Duane B. Huggett, Jon A. Arnot, Patrick N. Fitzsimmons, and Christina E. Cowan-Ellsberry. 2013. "Toward Improved Models for Predicting Bioconcentration of Well-Metabolized Compounds by Rainbow Trout Using Measured Rates of in Vitro Intrinsic Clearance." *Environmental Toxicology and Chemistry* 32(7):1611–22. doi: 10.1002/etc.2219.

OECD. 2018. "Test No. 280: Guidance Document on the Determination of in Vitro Intrinsic Using Cryopreserved Hepatocytes (RTHEP) or Liver S9 Sub-Cellular Fractions(RT-S9) from Rainbow Trout and Extrapolation to in Vivo Intrinsic Clearance Series on testing and Assessment." *OECD Guidel. Test. Chem.*

De Oro-Carretero, Paloma, and Jon Sanz-Landaluze. 2024. "In Vitro Approach to Refine Bioconcentration and Biotransformation Predictions of Organic Persistent Pollutants Using Cell Lines." *Chemosphere* 364. doi: 10.1016/j.chemosphere.2024.143020.

Ulrich, N. ,. Endo, S. ,. Brown, T. N. ,. Watanabe, N. ,. Bronner, G. ,. Abraham, M. H. ,. Goss, K. U. ,. 2017. "UFZ-LSER Database v3.2.1 [Internet]. Helmholtz Centre for Environmental Research-UFZ."
